# High-Fat Diet Exposure in Early Life Alters Mammary Metabolic and Inflammatory Microenvironment in Favor of Breast Tumorigenesis Later in Life in Mice

**DOI:** 10.3390/curroncol30040320

**Published:** 2023-04-17

**Authors:** Ying Tang, Ting-Chun Lin, Young-Cheul Kim, Soonkyu Chung, Zhenhua Liu

**Affiliations:** 1 Department of Nutrition, School of Public Health and Health Sciences, University of Massachusetts, Amherst, MA 01003, USA; yingtang@umass.edu (Y.T.); tingchunlin@umass.edu (T.-C.L.); yckim@nutrition.umass.edu (Y.-C.K.); soonkyuchung@umass.edu (S.C.); 2 UMass Cancer Center, University of Massachusetts Chan Medical School, Worcester, MA 01655, USA

**Keywords:** high-fat diet, obesity, early life, mammary microenvironment, inflammation, breast tumorigenesis

## Abstract

Emerging evidence highlights the important impact of early-life exposures on cancer development later in life. The present study aimed to investigate the impacts of a high-fat diet in early life on the mammary microenvironment in relation to breast tumorigenesis. Forty-four female C57BL/6 mice were fed a low-fat diet (LF, 10 kcal% fat) or a high-fat diet (HF, 60 kcal% fat) for 8 weeks starting at ~4 weeks of age. Twenty-two mice were sacrificed immediately after an 8 week feeding, and the rest of mice were switched to a normal diet for maintenance (Lab Diet, #5P76) for additional 12 weeks. A panel of metabolic parameters, inflammatory cytokines, as well as tumorigenic *Wnt*-signaling target genes were analyzed. The HF diet increased body weight and exacerbated mammary metabolic and inflammatory status. The disrupted microenvironment remains significant to the later life equivalent to young adulthood (*p* < 0.05). Mammary *Wnt*-signaling was elevated right after the HF diet as indicated by the upregulated expression of its downstream genes, whereas it was surprisingly suppressed after switching diets (*p* < 0.05). In summary, HF-induced overweight/obesity in early life altered the mammary metabolic and inflammatory microenvironments in favor of breast tumorigenesis, although its overall impact to breast cancer later in life warrants further investigation.

## 1. Introduction

Breast cancer (BC) is the most common cancer worldwide in women, contributing to a large portion of the global number of cancer deaths [1]. In the United States, approximately 330,250 new cases were diagnosed and ~43,250 women died from BC in 2022, and it represents the second leading cause of cancer deaths [2]. Although the increase in BC incidence in elder women is leveling off, emerging epidemiological evidence indicates a constant increase in BC among young women aged less than 40 years [3,4,5]. Adolescents and young adults (AYAs) aged 15–39 years are experiencing great challenges when confronting BC [3,6]. Therefore, understanding the risk factors for BC is crucial to combating this global health issue, particularly for AYAs.

Among evidential risk factors for BC, overweight and obesity are considered robust risk factors and are also associated with poor prognosis and outcome [7]. Overweight and obesity during adulthood have been shown to have a positive association with post-menopausal BC [8,9]. Studies have demonstrated several mechanisms governing this association. Obesity is associated with adverse remodeling of adipose tissue that predisposes one to metabolic dysregulation, and hypertrophic adipose tissue actively secretes adipokines and pro-inflammatory cytokines in favor of breast tumorigenesis [10,11]. In addition, the histological composition of breast tissue integrated with adipose tissue may specifically precipitate the estrogen-dependent growth and progression of BC cells in a paracrine manner, by which the steroid spreads from adipocytes to interact with estrogen receptors to promote cancer cell proliferation [12]. Elevated levels of sex steroids have been clearly demonstrated to be associated with the risk of postmenopausal BC among obese women [13].

Childhood obesity is a serious health concern in the United States. For children and adolescents aged 2–19 years in 2017–2020, the prevalence of obesity has reached 19.7%, accounting for 14.7 million children and adolescents [14]. About 55% of children with obesity will be obese in their adolescence, and around 80% of obese adolescents will continue to be obese in their adulthood [15]. Emerging evidence indicates early-life (during childhood, adolescence, or young adult years) events including obesity are critical risk factors for a variety of cancers later in life [16,17]. However, accumulating epidemiological studies have been showing that childhood/adolescent body fatness is surprisingly associated with a reduced risk of BC in both pre- and post-menopausal women [17,18,19]. The mechanism(s) responsible for this reverse relationship remain entirely unknown. The present study was specifically designed to characterize the impact of HF-induced obesity in early life, equivalent to childhood/adolescence in humans, on the mammary metabolic and inflammatory environment later in life, similar to young adulthood in humans.

## 2. Materials and Methods

### 2.1. Animals and Experimental Design

C57BL/6 mice were originally purchased from Jackson laboratory (Bar Harbor, ME, USA) and bred in our animal facility. Mice were housed under conditions of controlled temperature (22 °C–25 °C) and illumination (12:12 h light–dark cycle: lights on between 7:00 a.m. and 7:00 p.m.) with food and water provided ad libitum. The animal use protocol (#930) was approved by the Institutional Animal Care and Use Committee of the University of Massachusetts, Amherst.

**Figure 1:**
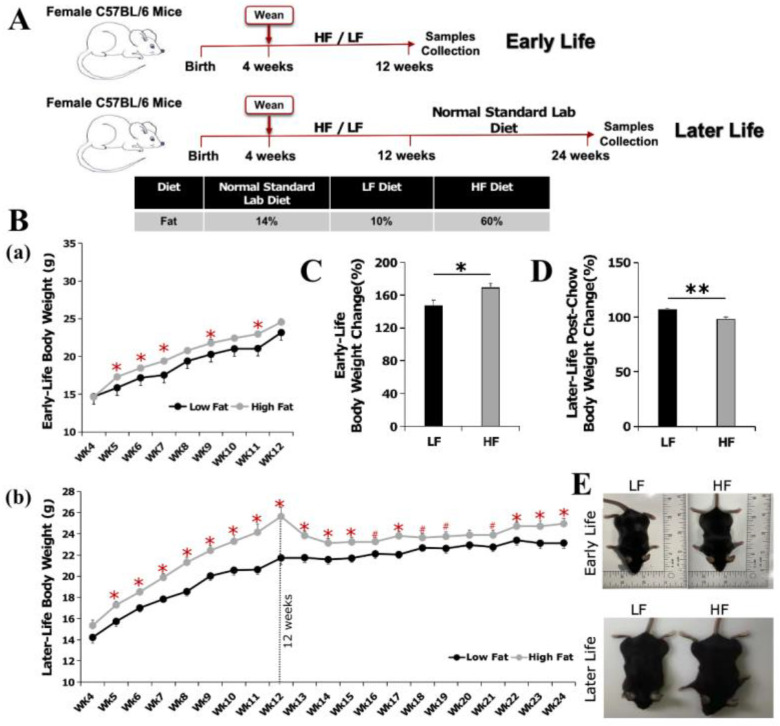
Experimental design and effect of high-fat diet in early life on body weight. (**A**) Experimental design: one half of the animals (22) were sacrificed immediately after the 8 week experimental HF/LF diet feeding at the age of ~12 weeks, which is equivalent to the end of adolescence in humans (Early Life), and another half were terminated after the 8 week HF/LF feeding plus a 12 week normal diet feeding at the age of ~24 weeks, which is equivalent to later in young adulthood (Later Life). (**B**) Growth curves of mice sacrificed (**a**) in early life after 8 week HF/LF feeding, and (**b**) in later life after 8 week HF/LF feeding + 12 week normal diet feeding. (**C**,**D**) Effect of high-fat diet in early life on body weight changes (%). (**E**) Representative pictures of mice sacrificed in early life and in later life. Data are presented as mean ± SEM. *n* = 10–11/group. #: 0.05 < *p* < 0.10; *: *p* < 0.05; **: *p* < 0.01.

The experimental design is shown in Figure 1A. According to the life history stages in C57BL/6J mice that are comparable to those in human beings [20,21], we fed experimental LF or HF diets only in the second and third month of age (~4–12 weeks of age), which is equivalent to the childhood and adolescence period in human. One half of the animals (22 animals) were sacrificed to determine the impact immediately after the 8 week experimental diet feeding during early life (Early Life). Another half of the animals were terminated after the 8 week HF/LF feeding plus another 12 week feeding with normal standard lab diet for growth and maintenance (LabDiet, Cat.#5P76, St. Louis, MO, USA, which is used in our animal facility) to define the impact of the early-life experimental diet on later-life microenvironment (Later Life), where 24 weeks of age in mice is equivalent to later in young adulthood in humans.

A standard high-fat diet for diet-induced obesity and a low-fat control diet from Research Diets (New Brunswick, NJ, USA) were used (HF: D12492, 60 kcal% fat vs. LF: D12450B, 10 kcal% fat). The HF diet contains 54% of its calories from lard, whereas only 4% of the calories are from lard in the LF. Mice were monitored daily (with one mouse dying during the experiment), and the body weight was measured weekly. After the 8 week experimental diet feeding (Early Life) or after the additional 12 week normal diet feeding (Later Life), mice were euthanized by CO2 asphyxiation followed by cervical dislocation. Exsanguination was performed by cardiac puncture and whole blood was collected into a blood collection tube containing EDTA (Govidien MonojectTM). After the abdomen was opened, inguinal mammary tissue (iMT) and visceral white adipose tissue (VAT) were collected into foil after removing the lymph nodes, and frozen in liquid nitrogen for subsequent biological assays.

### 2.2. Real-Time PCR for Gene Expression

Total RNA was extracted from inguinal mammary tissue and visceral white adipose tissue using the Trizol reagent (Invitorgen, Carlsbad, CA, USA). The concentration and purity of the RNA samples were determined using a NanoDrop 2000 (Thermo Scientific, Waltham, MA, USA). Subsequently, 2 µg total RNAs was reverse transcribed into the first-strand cDNAs using QuantiTect Reverse Transcription Kit (Qiagen, Valencia, CA, USA). Real-time PCR was performed with the ViiATM 7 Real-Time PCR System (Applied Biosystems, Carlsbad, CA, USA) using the SYBR green PCR reagent kit (Invitrogen, Carlsbad, CA, USA) and with the following thermal cycling conditions: 95 °C for 10 min, followed by 40 cycles of 95 °C for 15 s and 60 °C for 60 s. The cycle threshold (Ct) values were defined as the fractional cycle number at which the fluorescence passes an arbitrarily set threshold.

### 2.3. Western Blot Analyses

Protein was isolated from both inguinal mammary tissue and visceral white adipose tissue using RIPA lysis buffer (EMD Millipore Corp, Billerica, MA, USA) with Protease and Phosphatase inhibitor cocktail (Thermo Scientific, Rockford, IL, USA). The total protein concentration was determined using a commercially available PierceTM BCA Protein Assay Kit (Thermo Scientific, Waltham, MA, USA). Protein samples of 20–40 μg from each group were separated by either 8 or 10% SDS-PAGE gels and transferred onto a PVDF membrane. After blocking with 5% BSA in TBST solution, membranes were then probed with specific primary antibodies overnight at 4 °C, followed by incubation with the secondary antibody. The chemiluminescence from ClarityTM Western ECL Substrate (Bio-Rad, Hercules, CA, USA) was detected using an Odyssey FC Imaging System (Li-Cor). The band density was analyzed using Image J. The relative density of protein was calculated after normalizing to GAPDH.

### 2.4. Plasma and Mammary Inflammatory Cytokine and Metabolic Hormone Assays

The inflammatory cytokines and metabolic hormones were measured by a chemiluminescence assay using the QuickPlex SQ 120 (Meso Scale Discovery, Rockville, MD, USA). Assays were performed according to the manufacturer’s instructions. Briefly, the protein samples including inflammatory cytokines and metabolic hormones were isolated from inguinal mammary tissue with lysis buffer provided in the kit from Meso Scale Discovery (Rockville, MD, USA). Protein samples, plasma, or calibrator standards (25 µL) were added to each well of a 96-well plate. Antibodies for cytokines (TNF-α, IL-6, CCL2, and MCP-1) and metabolic hormones (Insulin, Leptin, and VEGF-A) were coated on the bottom of the 96-well plates. After washing 3 times, 50 µL of the detection antibody solution was added to each well. A four-parameter logistic fit curve was generated for each analyte using the standards, and the levels of inflammatory cytokines and metabolic hormones in the samples were calculated accordingly. Cytokines and metabolic hormones from inguinal mammary tissue are expressed as ng of analyte per mg protein. Cytokines and metabolic hormones from plasma are expressed as ng of analyte per milliliter. All standards and samples were measured in duplicate.

### 2.5. Statistical Analysis

All data were expressed as means ± SEM. For the statistical analysis, a two-tailed Student’s *t*-test was performed between the two treatment groups in both early life and later life using SAS (Version 9.4, SAS Institute, Cary, NC, USA). The gene expression was normalized to the housekeeping gene *GAPDH* (ΔCt = Ct-_Target Gene_ − Ct-*_GAPDH_*), and statistical analyses were performed based on ΔCt. Relative expression is reported as 2^−ΔΔCt^, where ΔΔCt = ΔCt-_Experiment_ − ΔCt-_Control_. Volcano plots were drawn in RStudio version 2022.12.0 (Rstudio, Boston, MA, USA) with ggplot2 and ggrepel packages. Values of *p* < 0.05 were considered statistically significant among the comparisons.

## 3. Results

### 3.1. High-Fat Diet Feeding in Early Life Increased Body Weight Gain, Which Is Retained at a Reduced Magnitude in Later Life of Young Adulthood

After an 8 week feeding during ~4–12 weeks of age, the HF diet significantly induced body weight gain (*p* < 0.05, Figure 1B(a),E). The body weight gains for the mice sacrificed immediately after the 8 week experimental diet feeding are 147% for LF group and 169% for HF group (Early Life, Figure 1C).

After switching from the HF diet to a normal diet for 12 weeks (up to 24 weeks of age), the body weight in the HF group remained significantly higher than that in the LF group (*p* < 0.05, Figure 1B(b),E), though the magnitude was reduced. The body weight increased by 7% (107%) for the LF group, whereas it decreased by 2% (98%) in the HF group when compared to the body weight immediately after the experimental diet feeding at the age of 12 weeks (Figure 1D).

### 3.2. Influence of Early-Life High-Fat Diet Feeding on Mammary Metabolic Microenvironment

#### 3.2.1. High Fat Diet in Early Life Promoted Adipocyte Dysfunction in Later Life

**Figure 2:**
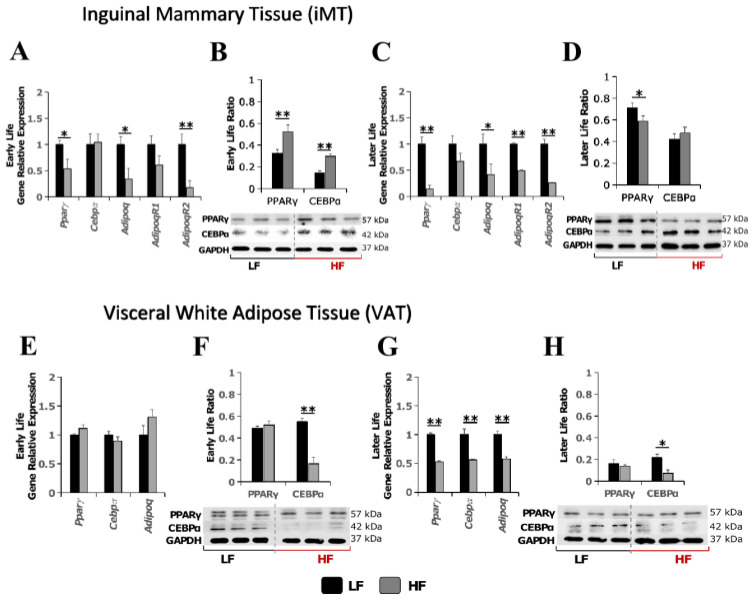
Effect of high-fat diet in early life on transcriptional and protein expressions of adipogenesis-related genes. (**A**–**D**) mRNA and protein expressions in inguinal mammary tissue in mice immediately after the 8 week experimental HF/LF diet feeding (Early Life, (**A**,**B**)) or later in young adulthood (Later Life, (**C**,**D**)); (**E**–**H**) mRNA and protein expressions in visceral white adipose tissue in mice immediately after the 8 week experimental HF/LF diet feeding (Early Life, (**E**,**F**)) or later in young adulthood (Later Life, (**G**,**H**)). Data are presented as mean ± SEM, *n* = 8–11/group. *: *p* < 0.05; **: *p* < 0.01.

To investigate the impact of the high-fat diet in Early Life on adipogenesis, we measured the expressions of a group of adipogenesis-related genes, including *Pparγ*, *Cebpα*, *Adiponectin* (*Adipoq*), *Adiponectin Receptor 1* (*AipoqR1*) and *Adiponectin Receptor 2* (*AipoqR2*), in iMT and VAT. In early life, immediately after the 8 week HF diet feeding, we observed a mixed pattern of mRNA and protein expressions of those genes in iMT and VAT. The protein level of PPARγ and CEBPα in iMT was higher (*p* < 0.01) in the LF group than in the HF group (Figure 2B), whereas this pattern was not observed in VAT (Figure 2F). Consistent results were observed for the mRNA and protein expressions of those genes in both iMT (Figure 2C,D) and VAT (Figure 2G,H) in the later life equivalent to young adulthood. The HF diet feeding during early life resulted in decreased expressions of the majority of those adipogenesis-related genes in later life, even after returning to a normal diet for 12 weeks.

#### 3.2.2. High-Fat Diet in Early Life Promoted Pro-Estrogenic Microenvironment in Later Life

**Figure 3:**
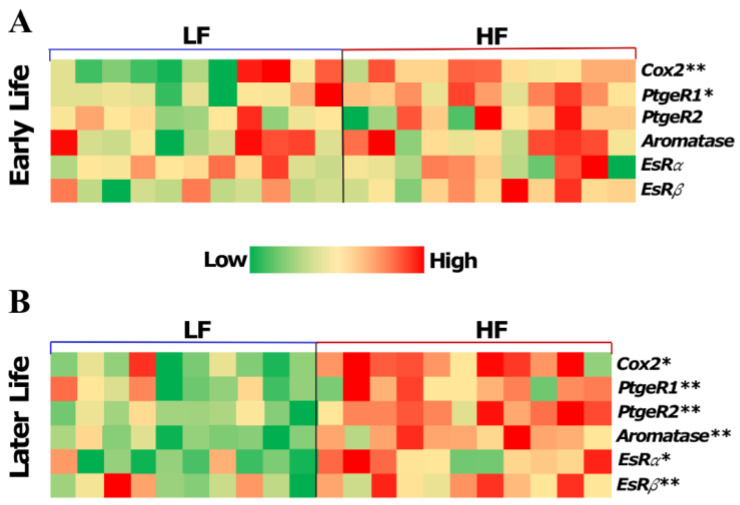
Effect of high-fat diet in early life on the expressions of hormone-related genes in the inguinal mammary tissues from mice sacrificed in early life immediately after the 8 week experimental HF/LF diet feeding at the age of ~12 weeks (**A**) and in later life after switching to the normal diet for an additional 12 weeks and at the age of ~24 weeks (**B**). Data are presented as mean ± SEM. *n* = 8–11 animals/group. *: *p* < 0.05; **: *p* < 0.01.

To investigate the impact of a high-fat diet in early life on the mammary hormonal microenvironment later in life, the transcriptional expression level of a panel of hormone-related regulators including *Aromatase*, *Estrogen Receptor α* and β (*Erα* and *Er*β), *Prostaglandin-endoperoxide synthase 2* (*Cox2*), and *Prostaglandin E2 receptors* (*PtgeR1* and *PtgeR2*) were measured in iMT. Immediately after the 8 week experimental diet feeding, the HF diet significantly increased the gene expression levels of *Cox2* and *PtgeR1* by 3.5-fold (*p* < 0.01) and 2.2-fold (*p* < 0.05), respectively, by the age of 12 weeks (Early Life, Figure 3A). After switching to a normal diet for an additional 12 weeks, the levels of *Cox2* and *PtgeR1* remained elevated. Furthermore, the increase in the expressions of *PtgeR2*, *Aromatase*, *ERα* and *ER*β reached a statistically significant level (Later Life, Figure 3B). The up-regulated hormone-related regulators indicated that the HF diet feeding during early life, equivalent to childhood and adolescence in humans, resulted in a promoted pro-estrogenic microenvironment in the period of later life equivalent to young adulthood.

### 3.3. High-Fat Diet in Early Life Instantly Induced a Pro-Inflammatory Microenvironment, whereas Switching to a Normal Diet Alleviated the Inflammatory Response

**Figure 4:**
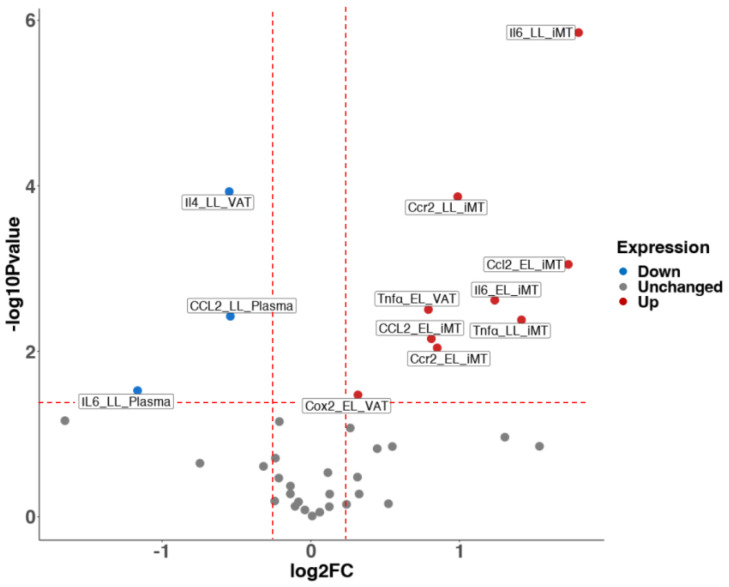
Volcano plot for the effect of high-fat diet in early life on inflammatory cytokine profile and metabolic parameters later in young adulthood. Red and blue points mark significantly increased or decreased expression, respectively, in HF (60 kcal % fat) vs. LF groups (10 kcal % fat) (*p* < 0.05). The *x*-axis shows log2(fold-changes) in expression and the *y*-axis shows the log10(*p*-value). Data represents mRNA or protein expressions in plasma and tissues. iMT: inguinal Mammary Tissue. VAT: Visceral White Adipose Tissue. EL: Early Life groups; LL: Later Life groups. *N* = 8–11 animals/group.

To investigate the impact of HF diet-induced obesity in early life on systemic (plasma) and mammary inflammation in later life, the mRNA and protein levels of inflammation-related genes, including *Tnf𝛼*, *Il-6*, *Ccl2*, *Ccr2*, *Il-1β*, *Cox2*, *Il-13*, and *Il-4*, were measured in plasma, iMT and VAT. In early life, immediately after the 8 week HF/LF diet feeding, we found that the HF diet is associated with 2.4-fold (*p* < 0.01), 3.3-fold (*p* < 0.01) and 1.8-fold (*p* < 0.01) increases in the transcriptional expression levels of *Il-6*, *Ccl2* and *Ccr2* in iMT, and that the protein level of CCL2 increased 1.8-fold (*p* < 0.01) in the HF group in iMT. The transcriptional expression levels of *Tnfα* and *Cox2* were found to be positively associated with HF diet, with 1.7-fold (*p* < 0.01) and 1.3-fold (*p* < 0.05) increases in VAT (Figure 4).

After switching to the normal diet for an additional 12 weeks up to the age of 24 weeks, the transcriptional expression levels of *Tnfα*, *Il-6* and *Ccr2* increased 2.7-fold (*p* < 0.01), 3.5-fold (*p* < 0.01) and 2.0-fold (*p* < 0.01) in HF diet group in iMT. However, the HF-induced systemic inflammation was marginally attenuated, as indicated by decreased protein levels of IL6 and CCL2, with 0.4-fold (*p* < 0.05) and 0.7-fold (*p* < 0.01) decreases in plasma and no significant difference between groups in VAT (Figure 4).

### 3.4. High-Fat Diet Merely Elevated the Expression of Wnt-Signaling Downstream Genes and Active Β-Catenin in Early Life

**Figure 5:**
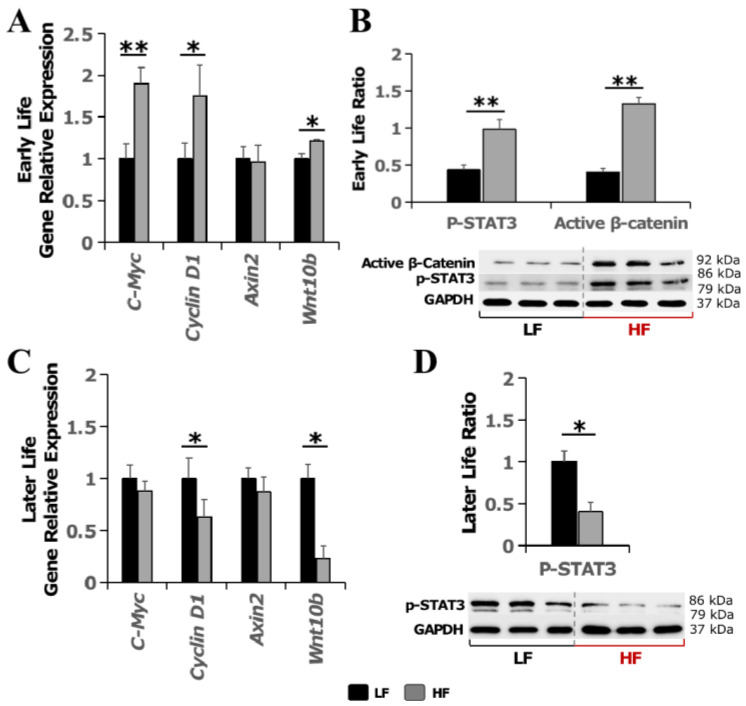
Effect of high-fat diet in early Life on *Wnt* signaling in inguinal mammary tissue later in life. (**A**) Transcriptional expression of *Wnt* signaling target genes and (**B**) Western blotting of phospho-STAT3 and active β-catenin in early life immediately after the 8 week experimental HF/LF diet feeding. (**C**) Transcriptional expression of *Wnt* signaling target genes and (**D**) Western blotting of phospho-STAT3 in later life after switching to the normal diet for an additional 12 weeks. Data are presented as mean ± SEM. *n* = 8–11 animals/group. *: *p* < 0.05; **: *p* < 0.01.

Aberrant Wnt signaling plays a critical role in mammary gland development and breast tumorigenesis [22,23]. STAT3 signaling possesses multifaceted functions in the immune response in the breast tumor microenvironment and has downstream target genes that overlap with the *Wnt* signaling pathway [24]. Therefore, we measured the transcriptional expression of *Wnt* signaling downstream genes (*Cmyc*, *Cyclin D1*, *Axin2*), *Wnt10b* and the protein level of p-STAT3 in iMT. Immediately after the 8 week experimental diet feeding, the HF diet increased transcriptional expression of *C-Myc* (*p* < 0.01), *Cyclin D1* (*p* < 0.05), and *Wnt10b* (*p* < 0.05) (Figure 5A) and the protein level of phosphor-STAT3 and active β-catenin (*p* < 0.01) (Figure 5B). Interestingly, after switching to the normal diet for an additional 12 weeks up to 24 weeks of age, which is equivalent to young adulthood, the transcriptional expression of *Cyclin D1* (*p* < 0.05) and *Wnt 10b* (*p* < 0.05) and the protein level of p-STAT3 had decreased in the HF group (Figure 5C,D).

## 4. Discussion

A global burden of disease study indicates that over 4 million people die each year as a result of being overweight or obese, a problem that has reached epidemic proportions [25]. In particular, the prevalence of obesity among children and adolescents continues to rise dramatically: from 2000 to 2018, the prevalence of obesity in persons aged 2–19 years increased from 13.9% to 19.3%, and the prevalence of severe obesity increased from 3.6% to 6.1% [26]. Childhood obesity is a risk for the development and mortality of a variety of cancers [27], whereas accumulating epidemiological studies have pointed out a negative association between childhood/adolescent obesity and both pre- and post-menopausal BC [17,18,19]. To the best of our knowledge, the present study is the first to use an animal model to mimic diet-induced obesity in the periods of early life equivalent to childhood and adolescence in humans, and examine its impact on the mammary microenvironment later in life. According to the lifespan comparison [21], the 8 week experimental diet feeding during 4-12 weeks of age in mice is equivalent to an HF intervention during childhood and adolescence in humans, and the experiment was finished at 24 weeks of age, which is equivalent to young adulthood in humans. Our findings, from the aspect of disrupted metabolic and inflammatory microenvironments, reinforced that obesity in early life remains a risk factor for the development of BC later in life.

In terms of the metabolic environment in iMT and VAT, we observed that the 8 week HF feeding in early life diminished the mRNA or protein expressions of a number of adipogenesis-related genes later in life (Figure 2C,D,G,H). PPARγ and CEBP𝛼 are two critical transcriptional factors, which cooperatively orchestrate adipocyte biology [28]. Emerging evidence indicates that PPARγ also controls cell proliferation in various other tissues and organs including the breasts, and the dysregulation of PPARγ signaling is linked to tumor development in these organs [29,30]. Adiponectin (Adipoq) is best known for modulating a number of metabolic processes including glucose homeostasis and fatty acid oxidation. In addition, adiponectin, via its receptors *AipoqR1* and *AipoqR2*, also plays a potent suppressive effect on the growth of breast cancer cells by cancer-specific fatty acid metabolic reprogramming [31]. The disrupted metabolic homeostasis, indicated by diminished expressions of adipogenesis-related genes such as *PPARγ* and *Adipoq* suggested that the HF-induced obesity in early life leads to adipogenesis dysfunction in iMT and potentially promotes breast tumorigenesis.

PPARγ has been shown to inhibit aromatase transcription, which is responsible for estrogen production via prostaglandin E2 (PGE2) [32,33]. Cyclooxygenase-2 (COX-2), which converts arachidonic acid to prostaglandins, also contributes to the production of aromatase in mammary tissue [34]. Aromatase is an enzyme responsible for a key step in the biosynthesis of estrogen, which is a critical risk factor for BC [35]. Findings from our work clearly demonstrated that HF feeding in early life led to the elevated expressions of these hormones in iMT (Figure 3), creating a hormonal microenvironment in favor of the development of tumorigenesis later in life.

Obesity is considered a low-grade chronic inflammatory status with the increased secretion of pro-inflammatory cytokines from adipocytes, including IL-6 and TNFα [36]. In addition, macrophages that have infiltrated into hypertrophic adipose tissue secrete an array of inflammatory cytokines, such as IL-1β, IL-6 and TNFα, contributing to both local and systemic inflammation [37]. Chronic inflammation plays a critical role in the initiation and development of BC [38]. In this study, we observed a systemic (plasma) and breast tissue-specific inflammation promoted by HF feeding in early life, evidenced by increased expression of TNFα, IL-6, CCL-2 and CCR-2 (Figure 4). This aggravated inflammation triggered by an HF diet in early life demonstrated that early-life obesity represents a promoting, rather than protecting, factor for the development of BC from the perspective of the inflammatory microenvironment in mammary tissue.

Early evidence for the involvement of the *Wnt* pathway in cancer came from the isolation of *Wnt-1*, a gene activated by the integration of the mouse mammary tumor virus in a mammary tumor model [39]. It has been reported that *Wnt* signaling abnormalities are associated with 60% of breast cancers [40,41]. Obesity-associated inflammation is linked to the up-regulation of active β-Catenin, a pivotal component in the *Wnt* pathway, and several *Wnt* signaling target genes (*Cyclin D1* and *Axin 2*) [42]. In early life, immediately after the 8 week HF feeding, our results are consistent with the aforementioned reports, demonstrating that the HF diet upregulated *Wnt* signaling target genes (*C-Myc* and *Cyclin D1*) and increased the protein levels of active β-Catenin as well as p-STAT3, an immune response gene that also regulates *Wnt* signaling target genes [24] (Figure 5A,B). However, in later life, to our surprise, we observed an opposite pattern of alterations of those *Wnt* signaling-related elements, which collectively indicate decreased *Wnt* signaling (Figure 5C,D). These observations could not be explained by the disrupted metabolic and promoted inflammatory microenvironment mediated by earlier exposure to the HF diet. Our group previously described a potential epigenetic mechanism, improved folate status and DNA methylation, responsible for the protective effect of early-life obesity on breast tumorigenesis [43]; however, further studies are needed to interpret the apparent protective effect of early-life obesity on BC later in life.

## 5. Conclusions

In summary, accumulating epidemiological studies have reported that overweight/obesity during childhood/adolescence may exert a protective effect on BC later in life. From the mammary metabolic and inflammatory microenvironment perspective, the present study showed that obesity in early life still imposes a risk on breast tumorigenesis later in life. However, further investigation is warranted to understand the overall impact of early-life obesity on breast tumorigenesis, and to therefore develop better strategies to manage obesity and BC.

## Data Availability

The data presented in this study are available in this article.
